# BTB and TAZ domain protein BT4 positively regulates the resistance to *Botrytis cinerea* in *Arabidopsis*

**DOI:** 10.1080/15592324.2022.2104003

**Published:** 2022-07-25

**Authors:** Fan Zhou, Kang Zhang, Xu Zheng, Guanyu Wang, Hongzhe Cao, Jihong Xing, Jingao Dong

**Affiliations:** State Key Laboratory of North China Crop Improvement and Regulation, Hebei Key Laboratory of Plant Physiology and Molecular Pathology, Hebei Agricultural University, Baoding, China

**Keywords:** *Arabidopsis thaliana*, *BT4*, *Botrytis cinerea*, disease resistance

## Abstract

*BT4* gene was identified to play an important role in *Arabidopsis* resistance to *pst* DC3000 in preliminary studies. However, the specific function and molecular mechanism of *BT4* gene in regulation of *Arabidopsis* resistance to *Botrytis cinerea* had not been described to date. In this study, we found that the expression of *BT4* was induced by wounding and *B. cinerea* inoculation in *Arabidopsis*. After inoculated with *B. cinerea*, T-DNA insertion mutants of the *BT4* gene, *bt4*, showed significant susceptibility symptoms, whereas no significant symptoms were found in wild-type (WT), the complemented transgenic plants (CE), and the overexpression transgenic plants (OE). After inoculated with *B. cinerea*, the expression levels of *JAR1* and *PDF1.2* genes in *bt4* mutant were induced; however, the expression levels of these genes in *bt4* mutant were significantly lower than those in the WT, CE, and OE. These results indicated that the *BT4* positively regulate the expression of genes in JA/ET signaling pathways. Therefore, the *BT4* may be involved in the regulation of JA/ET signaling pathways to affect *Arabidopsis* resistance to *B. cinerea*.

## Introduction

Gray mold caused by *Botrytis cinerea* is a world-wide plant disease, which seriously affects the yield and quality of crops. The isolation of resistant-related genes against *B. cinerea* and the molecular mechanism of its disease-resistant provide the theoretical basis for the guidance of disease control and research of the molecular mechanism of interaction between plants and pathogens. At present, a lot of resistance-related genes of *Arabidopsis* against *B. cinerea* were identified. These genes regulate the resistance of *Arabidopsis* to *B. cinerea* by regulating SA and JA/ET signaling pathways. ERF1 is the activation factor of JA/ET signaling pathway in *Arabidopsis*. Overexpression of *ERF1* could activate the expression of resistance-related genes *PDF1.2* and *ChiB* and increase resistance to *B. cinerea*.^1^ In *Arabidopsis MYB30* is a positive regulator of hypersensitive response (HR) in *Arabidopsis*. Overexpression of *MYB30* in tobacco and *Arabidopsis* were more resistant to *Pseudomonas syringae* pv. *tomato* DC3000 (*Pst* DC3000).^2^ *MYB46* could bound to the cis site of the promoter of cell wall peroxidase encoding gene *Ep5C* in *Arabidopsis* to regulate the synthesis of secondary cell wall in vascular bundles, which affected the resistance of *Arabidopsis* to *B. cinerea*.^3^ The *BOS1* (*MYB108*) gene is mainly involved in JA signaling pathway of *Arabidopsis*. Loss of *BOS1* displayed more sensitive to *B. cinerea* and increased the resistance to *Pst* DC3000.^4^ ERF19 as a novel player in the mitigation of PTI, and highlights a potential role for NINJA in fine-tuning ERF19-mediated regulation of *Arabidopsis* against *B. cinerea*.^5^ WRKY33 is a key transcriptional regulator of hormonal and metabolic processes toward *B. cinerea* infection and is essential for resistance.^6,7^

Approximately 80 BTB proteins were identified in Arabidopsis, and several reports showed that BTB protein play a variety of functions in organisms, including transcriptional activation and inhibition,^8^ regulation of cytoskeleton,^9^ regulation of ion channels,^10^ and ubiquitination of protein.^11^ Five BT proteins (BT1–BT5) with BTB and TAZ domains were grouped into a small subfamily that interacts with calmodulin. Among them, BT1, BT2, and BT4 were found to bind to bromo-domain transcriptional regulators and the BT2 promoter binds to transcription factor TELOMERASE ACTIVATOR1 (TAC1) to regulate telomerase activity in mature vegetative organs.^12,13^ Some genes containing BTB domains were involved in plant disease resistance, such as NPR1 of Arabidopsis.^14^

Previously, the *BT4* gene, encoding a transcriptional regulator with BTB (broad-complex, tramtrack, and bric-a-brac) and TAZ (a transcriptional adapter zinc finger) domains, was isolated and identified as a resistant-related gene in response to *Pst* DC3000; meanwhile, *BT4* also could be induced by SA and JA^15^ . In this study, we investigated the function of *BT4* in *Arabidopsis* resistance to *B. cinerea* and further analyzed the probable mechanism of *BT4* gene regulation in *Arabidopsis* resistance to *B. cinerea*.

## Results

### *BT4* positively regulates the resistance to *B.* *cinerea* in *Arabidopsis*

**Figure 1.**
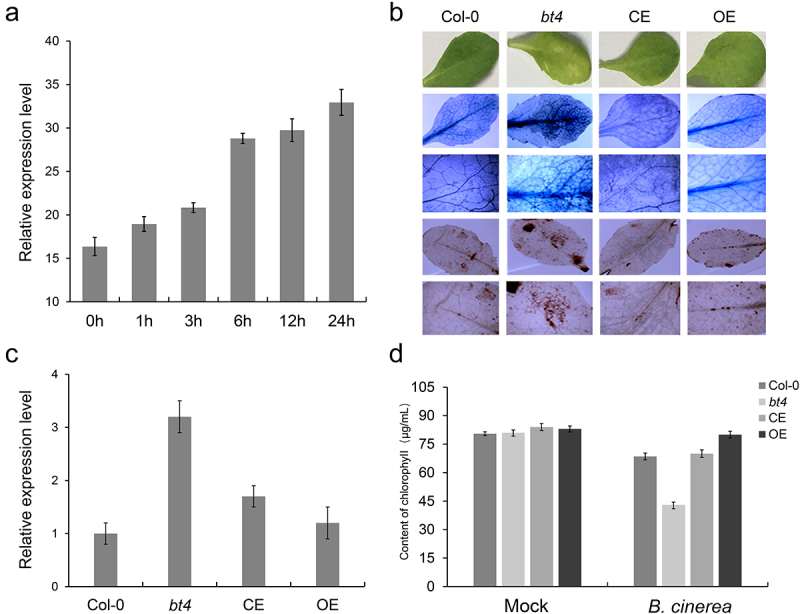
**The *BT4* gene positively regulate the resistance of *Arabidopsis* to *Botrytis cinerea***. (a) The expression level of *BT4* gene under wounding treatment. (b) Trypan blue and DAB observation of Col-0, *bt4* mutant, CE and OE. (c) Expression level *BcACTIN* in Col-0, *bt4* mutant, CE and OE inoculated by *B. cinerea*. (d) Evaluation of Col-0, *bt4* mutant, CE and OE chlorophyll content after inoculation of *B. cinerea.*

**Figure 2.**
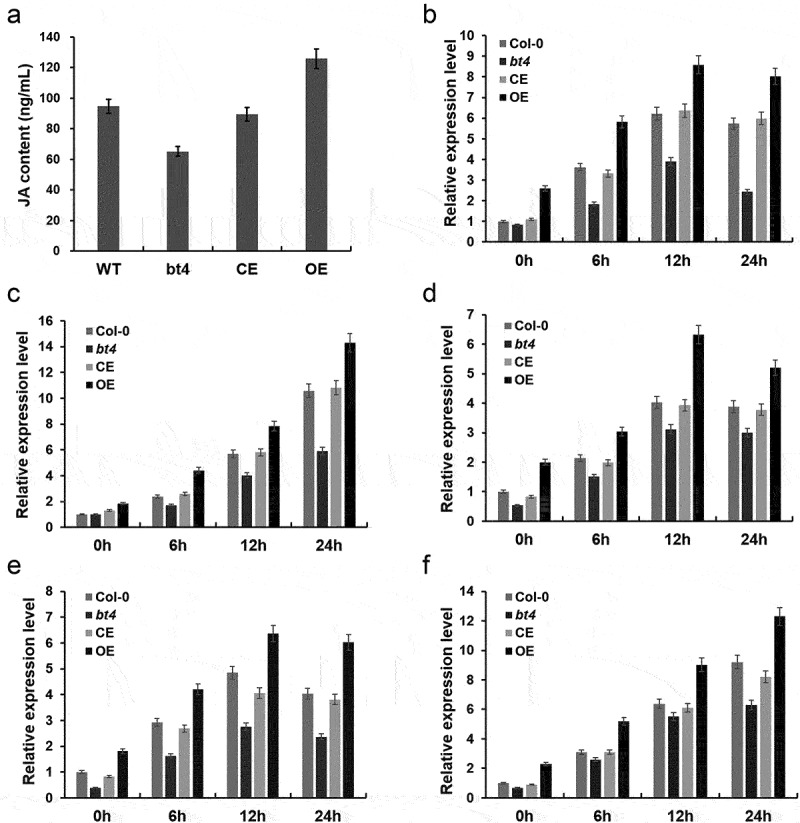
**The expression of resistance-related genes inoculated with *Botrytis cinerea***. (a) Contents of JA in Col-0, *bt4* mutant, CE and OE. (b) The expression level of *JAR1* inoculated by *B. cinerea* in different timepoint. (c) The expression level of *PDF1.2* inoculated by *B. cinerea* in different timepoint. (d) The expression level of *PR3* inoculated by *B. cinerea* in different timepoint. (e) The expression level of *JAL35* inoculated by *B. cinerea* in different timepoint. (f) The expression level of *LOX2* inoculated by *B. cinerea* in different timepoint.

Wounding provides nutrients to pathogens and facilitates their entry into the tissue and subsequent infection. Plants have evolved constitutive and induced defense mechanisms to properly respond to wounding and prevent infection. To explore the expression level of BT4 under wounding stress, we analyzed the datasets related to wounding stress from the GEO database (GSE101422). It was found that BT4 was significantly induced by wounding across different timepoint (Figure 1a). To study the function of the *BT4* gene in *Arabidopsis* resistance to *B. cinerea*, the Col-0, *bt4*, CE, and OE plants were inoculated with *B. cinerea* to investigate the resistance of mutants to *B. cinerea*. The results have shown that *bt4* mutant exhibited enhanced susceptibility to *B. cinerea*, whereas the Col-0, CE and OE plants showed obvious resistance to *B. cinerea* (Figure 1b). Trypan blue and DAB staining revealed that there were numerous dead cells and ROS in the leaves of the *bt4* mutant inoculated with *B. cinerea*, with fewer dead cells and ROS were observed in leaves of Col-0, CE and OE plants (Figure 1b). Consistent with this phenotype, the expression levels of *BcACTIN* were significantly up-regulated in *bt4* mutant (Figure 1c), and the content of chlorophyll were decreased obviously in *bt4* mutant (Figure 1d). In addition, the plant hormone JA in *bt4* mutants were also detected, and it was found that the contents of JA were also affected by the expression of *BT4* gene. The contents of the plant hormone JA were significantly reduced in *bt4*, partially recovered in CE, and the highest in OE, exceeding Col-0 (Figure 2a). The results showed that *bt4* mutant had strong susceptibility to *B. cinerea*, and indicated that the *BT4* gene played a positive role in *Arabidopsis* resistance to *B. cinerea*.

### *BT4* deficiency alters the expression of key JA signaling pathway genes in response to *B.* *cinerea*

To investigate whether the mutation of the *BT4* gene affects the expression of key genes in JA signaling pathways, we further detected the expression level of *JAR1* (Jasmonic acid-amido synthetase), *PDF1.2* (Plant defensin 1.2), *PR3* (Pathogenesis-related 3), *JAL35* (Jacalin-related Lectin 35) and *LOX2* (lipoxygenase 2) in the Col-0, *bt4*, CE, and OE plants inoculated with *B. cinerea*. The expression levels of *JAR1, PDF1.2*, PR3, *JAL35* and *LOX2* were obviously reduced by *B. cinerea* in the Col-0, *bt4*, CE, and OE plants (Figure 2(b-f)). However, the expression levels of these genes were significantly down-regulated in *bt4* mutants compared to those in the Col-0, CE, and OE plants. These results suggested that the *BT4* gene might affect the expression of JA/ET signaling pathway genes in response to *B. cinerea* infection.

## Conclusion

The JA signaling pathways play critical roles in protecting plant against pathogens.^16^ JA and ET are also involved in induced systemic resistance of plants.^17^ In this study, we found that wounding could induce the expression level of *BT4* gene. After inoculation with *B. cinerea*, the expression levels of key genes of the JA signaling pathways in the *bt4* mutant were significantly lower than those in the wild-type, CE, and OE mutant. These results indicated that the *BT4* gene might be involved in the regulation of JA signaling pathways and further regulated the resistance to *B. cinerea* in *Arabidopsis*.

## Materials and methods

### Plant materials and growth conditions

*Arabidopsis* wild-type Col-0, *BT4* gene T-DNA insertion mutant *bt4* (SALK-015577), *BT4* gene complemented transgenic plants CE, and the *BT4* gene overexpression transgenic plants OE were provided by Mycotoxin and Molecular Plant Pathology Laboratory (Hebei Agricultural University, Baoding, China). *Arabidopsis* were grown on mixture of vermiculite: plant ash: perlite (6:2:1) in a greenhouse with a rhythm of 16 h light/8 h dark.

### *B.* *cinerea* inoculation

*B. cinerea* (B05.10), provided by Hebei Key Laboratory of Plant Physiology and Molecular Pathology (Hebei Agricultural University, Baoding, China), was grown on PDA media and incubated at 22°C for 2 weeks. The conidia suspension of *B. cinerea* was prepared by brushing the *B. cinerea* medium with distilled water. The conidia suspension of *B. cinerea* was filtered and adjusted the final concentration of 4 ~ 8 × 10^6^ conidia/ml. Four-week-old of *Arabidopsis* plants were used to inoculate with 5 μl conidia suspension of *B. cinerea* placed onto individual leaves.

Inoculated *Arabidopsis* plants were moisturized at 22°C for 24 h in darkness and then incubated under normal conditions. At 0, 3, 6, 12, and 24 h post-inoculation (hpi), *Arabidopsis* plants were collected and used for Real-time PCR analysis. At 48 hpi, lesion formation was observed and the inoculated leaves were stained with trypan blue and DAB, and used for and relative expression level of *B. cinerea BcACTIN* and determination of chlorophyll content.

### RNA extraction and real-time PCR analysis of gene expression

Total RNA of *Arabidopsis* plants were extracted with Trizol regent (TaKaRa, Dalian, China) according to the manufacturer’s instructions and treated with RNase-free DNase I (Omega, USA) to remove genomic DNAs. The first strand cDNA was obtained using PrimeScript RT regent kit (TaKaRa, Dalian, China). The Real-time PCR reaction was done on a CFX96 real-time PCR system (BioRad, Hercules, CA, USA) in 10 μL reactions containing 0.2 μL SYBR Premix Ex Taq^TM^ (TaKaRa, Dalian, China) and each gene-specific primer (Table S1). Relative gene expression levels were calculated using 2^−CT^ method with three independent biological replicates.

## Supplementary Material

Supplemental Material
