# Supplementary material for: BTB and TAZ domain protein BT4 positively regulates the resistance to Botrytis cinerea in Arabidopsis
Source: Plant Signal Behav. 2022 Jul 25;17(1):2104003. doi: 10.1080/15592324.2022.2104003 (PMC9318297; doi:10.1080/15592324.2022.2104003)
Supplement: Supplemental Material [file KPSB_A_2104003_SM3852.docx]

**Table S1. Quantitative real-time PCR primers used in this study.**

| Gene name | Primer sequence |
| --- | --- |
| *BT4* | CCGAACCATGCAACGTGTA  TGCAATGCACGCAACCTCCTG |
| *JAR1* | CAACTAGCGCAGGATGTTGG  AATGGCGGATTGGTTCTTGAG |
| *PDF1.2* | TCTCTTTGCTGCTTTCGACG  TGTTTCCGCAAACCCCTGAC |
| *PR3* | ACTCAACAACCCTGACCTT |
|  | CGTAATCACTCCATAACCC |
| *JAL35* | TTCCAATACTATTCCAGCAC |
|  | ACGGCAGTGACATACTCA |
| *LOX2* | TACATAATAGCGGCAAATAG |
|  | GTAAGCCTTCCTGGTCAA |
| *18s rRNA* | TCGTAGTTGAACCTTGGGATGG  GACCCGGCCAATTAAGACCAG |
| *BcACTIN* | CTACTTTTGGTCGTTTGGTTCAC  ATGCAGCATAATCTCTTCACAAC |
